# Ionic dynamics of the cation in organic–inorganic hybrid compound (CH_3_NH_3_)_2_MCl_4_ (M = Cu and Zn) by ^1^H MAS NMR, ^13^C CP MAS NMR, and ^14^N NMR

**DOI:** 10.1039/c8ra02363h

**Published:** 2018-05-22

**Authors:** Ae Ran Lim

**Affiliations:** Analytical Laboratory of Advanced Ferroelectric Crystals, Jeonju University Jeonju 55069 Korea aeranlim@hanmail.net arlim@jj.ac.kr +82-063-220-2053 +82-063-220-2514; Department of Science Education, Jeonju University Jeonju 55069 Korea

## Abstract

The ionic dynamics of (CH_3_NH_3_)_2_MCl_4_ (M = Cu, Zn) by ^1^H magic-angle spinning (MAS) nuclear magnetic resonance (NMR), ^13^C cross-polarization (CP) MAS NMR, and ^14^N NMR are investigated as a function of temperature with a focus on the role of the CH_3_NH_3_^+^ cation. The molecular motions in (CH_3_NH_3_)_2_MCl_4_ are also discussed based on the ^1^H spin–lattice relaxation time in the rotating coordinate frame *T*_1ρ_. From the ^1^H *T*_1ρ_ results, the activation energies for the tumbling motion of ^1^H for CH_3_ and NH_3_ were similar, and the uniaxial rotations occurred within a large temperature range. The molecular motions for ^13^C and ^14^N of the main chain in the CH_3_NH_3_^+^ cation were rigid, whereas those for ^1^H of the side chain in the CH_3_NH_3_^+^ cation were very free at high temperatures. *T*_1ρ_ provides insight into the changes in the cation reorientation rates induced by heating at high temperatures.

## Abstract 2

The ionic dynamics of (CH_3_NH_3_)_2_MCl_4_ (M = Cu, Zn) by ^1^H MAS NMR, ^13^C CP MAS NMR, and ^14^N NMR are investigated as a function of temperature with a focus on the role of the CH_3_NH_3_^+^ cation.

## Graphical abstract

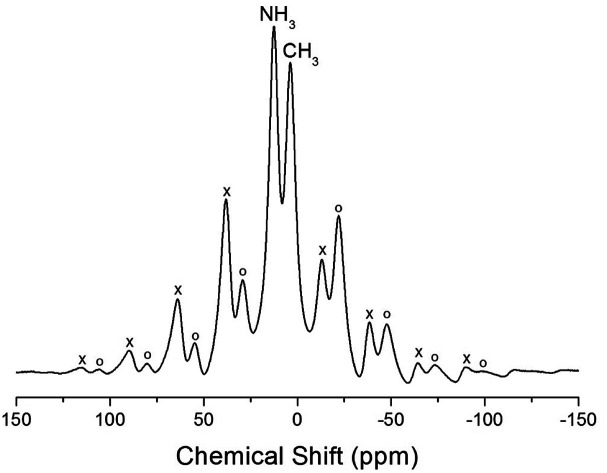

## 1. Introduction

Hybrid organic–inorganic compounds have been known since 1976 but recently they have been revisited due to their potential use as substitute materials for perovskites.^1–6^ Metal complexes with the formula (CH_3_NH_3_)_2_MCl_4_ (M = Mn, Fe, Cu, Zn, Cd) can be classified into two groups from a crystal structure point of view.^7–14^ One group (CH_3_NH_3_)_2_MCl_4_ (M = Mn, Fe, Cd) has a perovskite-type layer structure consisting of cationic layers and layers of corner-sharing chlorine octahedra with a divalent metal ion at the center.^15–18^ These compounds are characterized by a two-dimensional metal–chlorine network widely separated from one another by methyl ammonium groups. The metal ions are surrounded by a slightly distorted chlorine octahedron, Cl_6_. The other group, to which (CH_3_NH_3_)_2_MCl_4_ (M = Cu, Zn) belongs, consists of discrete CH_3_NH_3_^+^ and MCl_4_^2−^ ions packed in an arrangement similar to orthorhombic K_2_SO_4_-like members.^19,20^ In these crystals, unassociated Cl_4_ tetrahedra are presented instead of corner-sharing layers of chlorine octahedra. Hydrogen-bonding takes place between the hydrogens of CH_3_NH_3_^+^ and Cl^−^, and the several different possible hydrogen-bond configurations can give rise to structural phase transitions.

The (CH_3_NH_3_)_2_CuCl_4_ compound with M = Cu undergoes a structural phase transition at 348 K (= *T*_C_), with the respective phases denoted as orthorhombic structure at high temperature and monoclinic structure at room temperature.^21^ A sharp peak at 230 K from a thermal capacity experiment was also reported by White and Staveley.^22^ In the case of (CH_3_NH_3_)_2_ZnCl_4_ with M = Zn, the existence of a phase transition at 483 K (= *T*_C_) was reported by calorimetric, dielectric, thermal expansion, and optical measurements.^23^ However, a transition at 426 K 
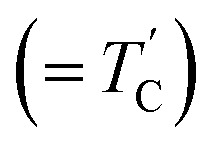
 was reported from Raman and IR spectra but not by differential scanning calorimetry (DSC), differential thermal analysis (DTA), and ^1^H nuclear magnetic resonance (NMR) measurements. The structure of (CH_3_NH_3_)_2_ZnCl_4_ is orthorhombic at high temperatures and monoclinic at low temperatures. In addition, it has been reported from low-temperature DSC that a phase transition exists at 265 K during heating.^24,25^

Following previous NMR investigations, the spin–lattice relaxation time *T*_1_ of ^1^H in the CH_3_ and NH_3_ groups of (CH_3_NH_3_)_2_CuCl_4_ at the Larmor frequencies of 12 and 26 MHz was reported. The spectra of the two groups overlap at high temperatures and separate at low temperatures.^25^ The *T*_1_ at low temperatures exhibits a strong temperature dependence. Moreover, the self-diffusion and reorientation of the methylammonium ions in (CH_3_NH_3_)_2_ZnCl_4_ was reported by ^1^H NMR.^26^ In addition, the spin–spin relaxation time *T*_2_ of ^63^Cu and ^35^Cl in (CH_3_NH_3_)_2_CuCl_4_ has been reported at 1.75 K.^27,28^ In the case of (CH_3_NH_3_)_2_ZnCl_4_, ^1^H *T*_1_ NMR studies at the Larmor frequency of 20 MHz revealed that the cation in the highest-temperature phase performs isotropic rotation and self-diffusion. The cation in the low-temperature phase undergoes reorientation about its C–N bond axis.^29^ Although the structural phase transitions in (CH_3_NH_3_)_2_CuCl_4_ and (CH_3_NH_3_)_2_ZnCl_4_ have been performed by several research groups, the corresponding molecular motions and structural geometry changes have not been fully studied by NMR in the rotating frame.

In the present study, to clarify the ionic dynamics of CH_3_NH_3_^+^ cations and to also obtain information of the mechanism of the phase transition in (CH_3_NH_3_)_2_MCl_4_ (M = Cu, Zn), the chemical shifts and spin–lattice relaxation time in the rotating coordinate frame *T*_1ρ_ were measured as a function of temperature using ^1^H magic-angle spinning (MAS) NMR and ^13^C cross-polarization (CP) MAS NMR. In addition, the ^14^N NMR spectra in (CH_3_NH_3_)_2_ZnCl_4_ single crystals in the laboratory frame were discussed in order to elucidate the structural geometry. We focus on the structural phase transitions of compounds with the formula (CH_3_NH_3_)_2_MCl_4_. We use these results to analyze the behavior of CH_3_ and NH_3_ near the phase transition temperature from the results of ^1^H MAS NMR, ^13^C CP MAS NMR, and ^14^N NMR. In addition, we compare the structural geometries of (CH_3_NH_3_)_2_MCl_4_ (M = Cu, Zn) obtained here and (CH_3_NH_3_)_2_MCl_4_ (M = Mn, Cd) previously reported.

## 2. Materials and methods

### 2.1. Crystal structure

**Fig. 1:**
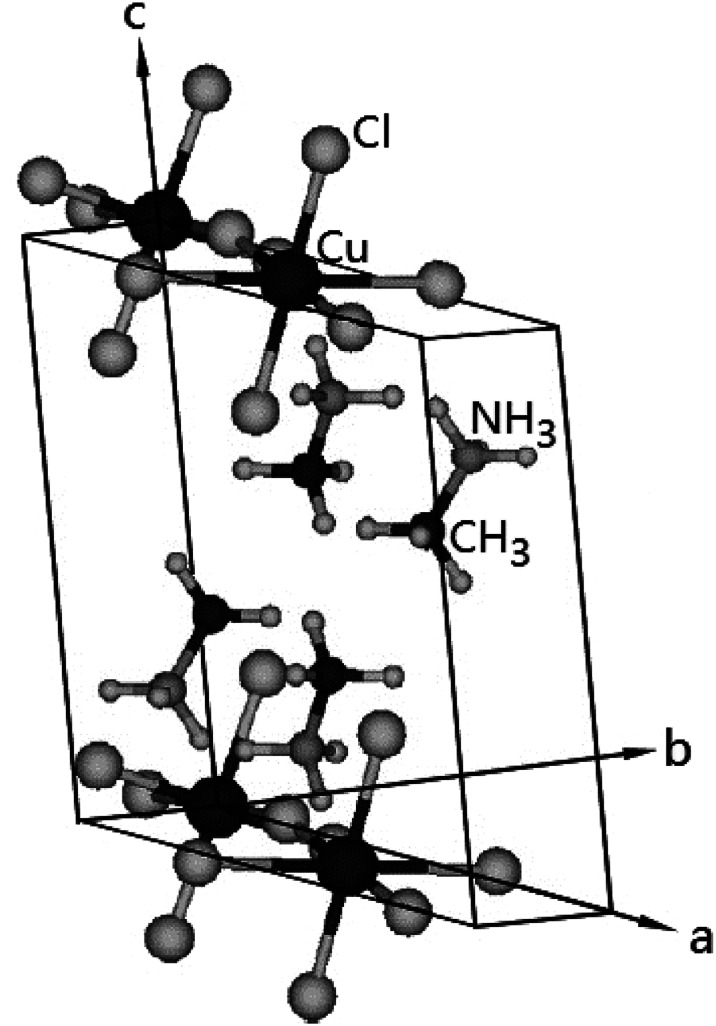
The structure of (CH_3_NH_3_)_2_CuCl_4_ at room temperature.

The (CH_3_NH_3_)_2_CuCl_4_ undergoes a phase transition at 348 K. At temperatures below *T*_C_ = 348 K, the structure is monoclinic, the space group is *P*2_1_/*c*, and the lattice constants are *a* = 7.155 Å, *b* = 7.424 Å, *c* = 9.814 Å, and *β* = 109.18°.^6^ The crystal structure at 363 K is orthorhombic, the space group is *Ccmb*, and the lattice constants are *a* = 7.34 Å, *b* = 18.71 Å, and *c* = 7.33 Å.^30,31^ The monoclinic structure at room temperature is shown in Fig. 1.^3^ Here, the methylammonium moieties are located between the layers and are connected by hydrogen bonds to the Cl^−^ ions. Further, (CH_3_NH_3_)_2_ZnCl_4_ undergoes a phase transition at 483 K. At room temperature, the crystal is monoclinic with the space group *P*2_1_/*c*, and the lattice constants are *a* = 10.873 Å, *b* = 12.655 Å, *c* = 7.648 Å, *β* = 96.71°, and *Z* = 4.^19,23,26,32^ Here, the two inequivalent sites, CH_3_(1) and CH_3_(2), and NH_3_(1) and NH_3_(2), in (CH_3_NH_3_)_2_ZnCl_4_ were reported by Morosin *et al.*^19^

### 2.2. Experimental method

Single crystals of (CH_3_NH_3_)_2_MCl_4_ (M = Cu, Zn) were prepared by the slow evaporation of an aqueous solution of stoichiometric amounts of CH_3_NH_3_·HCl and MCl_2_ (M = Cu, Zn) at room temperature. The color of (CH_3_NH_3_)_2_CuCl_4_ single crystals is brown with flat parallelepipeds. In addition, (CH_3_NH_3_)_2_ZnCl_4_ single crystals are colorless and transparent with a square shape. The phase transition temperatures were determined using DSC (Dupont, 2010) measurements at a heating rate of 10 K min^−1^.

^1^H MAS NMR and ^13^C CP MAS NMR spectra of (CH_3_NH_3_)_2_MCl_4_ (M = Cu, Zn) in the rotating coordinate frame were obtained at the Larmor frequencies of *ω*_0_/2π = 400.13 and 100.61 MHz, respectively, using a Bruker 400 MHz NMR spectrometer at the Korea Basic Science Institute, Western Seoul Center. Powdered samples were placed in a 4 mm CP MAS probe, and the MAS rate was set to 10 kHz for both ^1^H MAS and ^13^C CP MAS measurements to minimize the spinning sideband overlap. The chemical shifts were referred with respect to tetramethylsilane (TMS). The spin–lattice relaxation times for ^1^H and ^13^C of (CH_3_NH_3_)_2_MCl_4_ in the rotating coordinate frame were determined using a π/2 − *t* sequence by varying the duration of the spin-locking pulses. In the case of (CH_3_NH_3_)_2_CuCl_4_, the width of the π/2 pulse used for measuring the *T*_1ρ_ values of ^1^H and ^13^C was 3.9 μs, with a spin-locking field of 64.1 kHz. In the case of (CH_3_NH_3_)_2_ZnCl_4_, the width of the π/2 pulse used for measuring the *T*_1ρ_ values of ^1^H and ^13^C was 4.5 and 5.6 μs, with the spin-locking field of 55.55 kHz and 44.64 kHz, respectively. The power level for ^1^H and ^13^C was 4 db and 6.5 db, respectively. The ^13^C *T*_1ρ_ values were measured by varying the duration of the ^13^C spin-locking pulse applied after the CP preparation period.

In addition, the ^14^N NMR spectra of the (CH_3_NH_3_)_2_ZnCl_4_ single crystals in the laboratory frame were measured using a Unity INOVA 600 NMR spectrometer at the same facility. The static magnetic field was 14.1 T and the Larmor frequency was set to *ω*_0_/2π = 43.342 MHz. The ^14^N NMR experiments were conducted using a solid-echo pulse sequence.

Temperature-dependent NMR spectra were recorded at 180–430 K as the chemical shift and relaxation time could not be determined outside this temperature range, because of the limitations of the spectrometer used. The sample temperatures were maintained within ±0.5 K by controlling the nitrogen gas flow and heater current.

## 3. Results and discussion

**Fig. 2:**
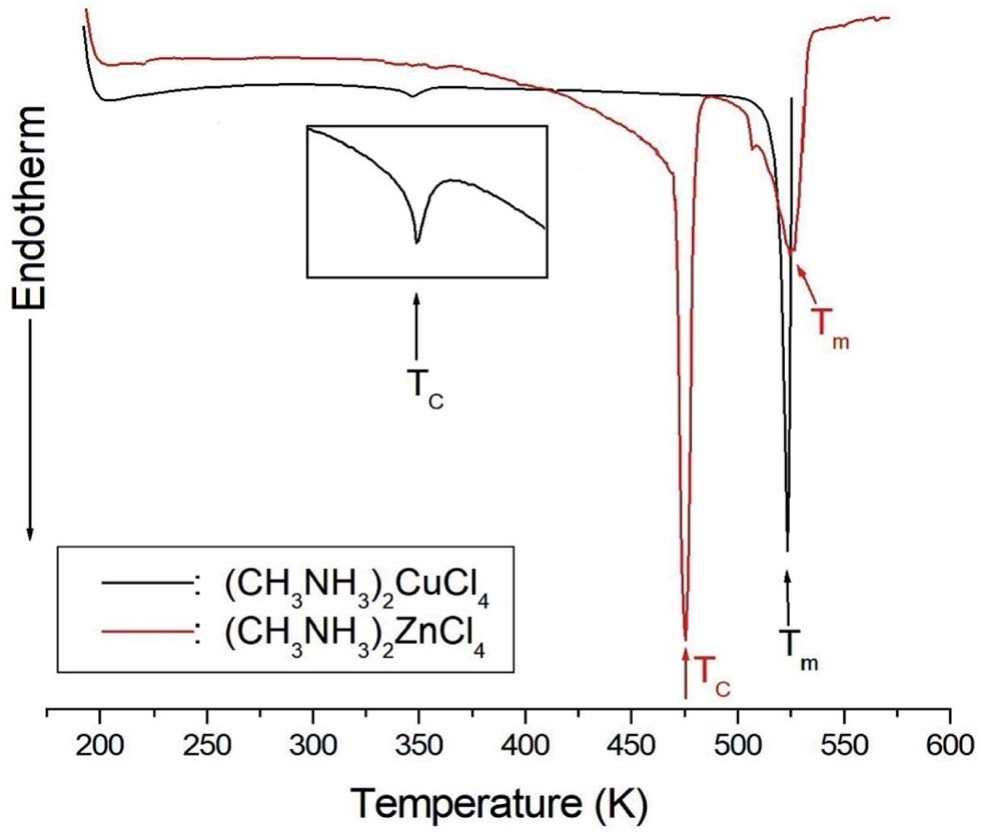
Differential scanning calorimetry thermogram of (CH_3_NH_3_)_2_CuCl_4_ and (CH_3_NH_3_)_2_ZnCl_4_ single crystals.

The DSC analysis in (CH_3_NH_3_)_2_CuCl_4_ revealed two endothermic peaks at 347 K (= *T*_C_) and 517 K (= *T*_m_) related to the phase transition and melting point, respectively, as shown in Fig. 2. The enlarged peak near 347 K in Fig. 2 is very small relative to the other endothermic peak. In the case of (CH_3_NH_3_)_2_ZnCl_4_, two endothermic peaks are obtained at 475 K (= *T*_C_) and 525 K (= *T*_m_), which are due to the phase transition and melting point. In order to understand the additional endothermic peaks at high temperature, we conduct optical polarizing microscopy. The peaks of 517 and 525 K in (CH_3_NH_3_)_2_CuCl_4_ and (CH_3_NH_3_)_2_ZnCl_4_, respectively, are not related to physical changes such as structural phase transitions; they are instead related to the melting point. The phase transition temperatures obtained here are consistent with previous results.^21,23^ This suggests that the differences in the chemical properties of Cu and Zn are responsible for the variations of the phase transition temperatures *T*_C_ in the two crystals.

**Fig. 3:**
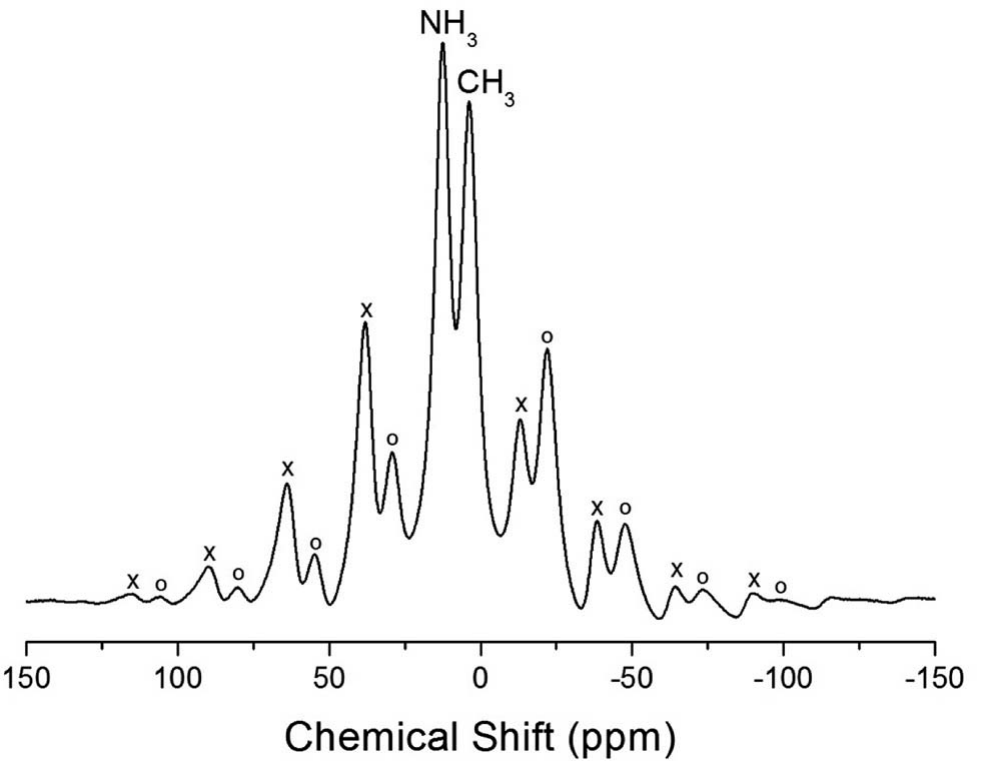
^1^H MAS NMR spectra of (CH_3_NH_3_)_2_CuCl_4_ at 300 K (the spinning sidebands are marked with crosses and open circles).

**Fig. 4:**
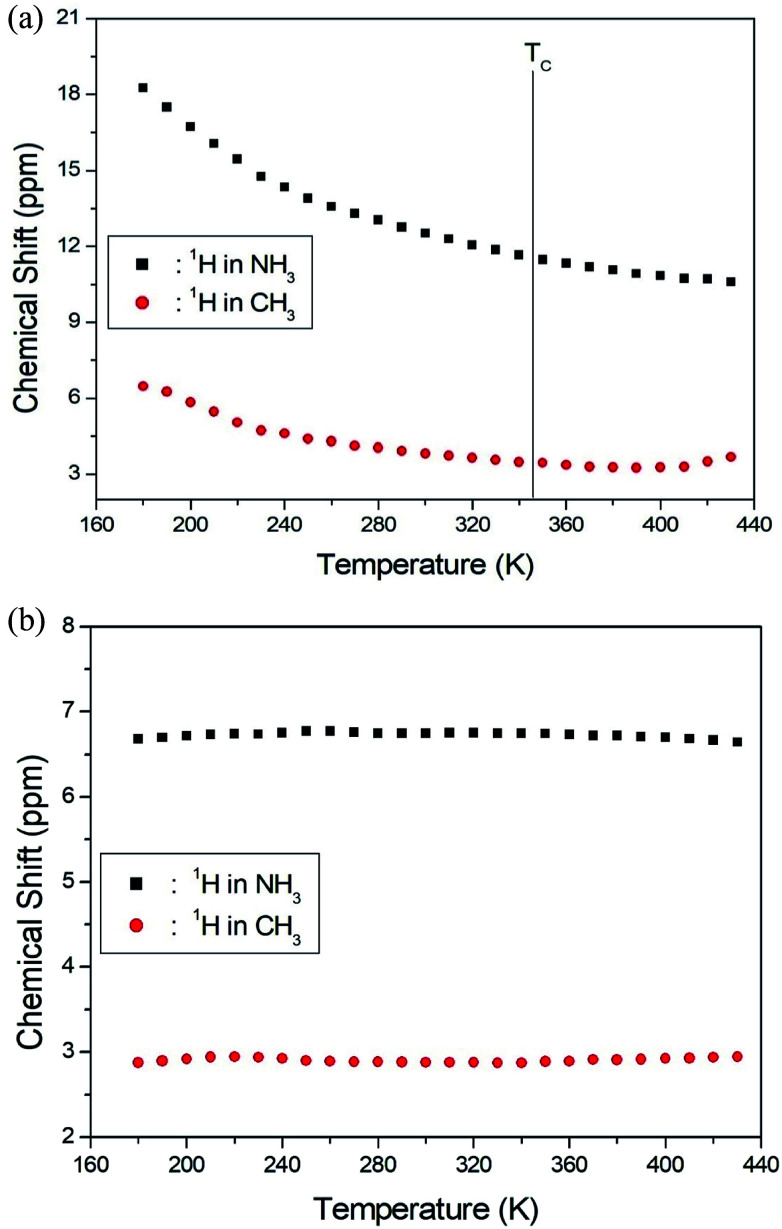
(a) ^1^H chemical shifts for CH_3_ and NH_3_ groups in (CH_3_NH_3_)_2_CuCl_4_ as a function of temperature. (b) ^1^H chemical shifts for CH_3_ and NH_3_ groups in (CH_3_NH_3_)_2_ZnCl_4_ as a function of temperature.

The NMR spectra for ^1^H in (CH_3_NH_3_)_2_MCl_4_ (M = Cu, Zn) were recorded by MAS NMR at a frequency of 400.13 MHz. In the case of the two compounds, the spectrum of the two peaks is assigned to the ^1^H in CH_3_ and NH_3_. One of them, the spectrum of the two peaks at chemical shifts of *δ* = 3.82 and 12.52 in (CH_3_NH_3_)_2_CuCl_4_ at room temperature, is presented in Fig. 3. Here, the unit of the NMR scale is represented according to the IUPAC convention.^33,34^ The spinning sidebands for CH_3_ are marked with open circles and those for NH_3_ are marked with crosses. The line component of the lower chemical shift is attributed to the ^1^H in CH_3_, and that of the higher chemical shift is attributed to the ^1^H in NH_3_. The protons of CH_3_ and NH_3_ are distinguished from the ^1^H chemical shifts. In the case of (CH_3_NH_3_)_2_CuCl_4_ across the phase transition temperature of *T*_C_, the chemical shift slowly and monotonously decreases with temperature, indicating that the environments of the surrounding ^1^H in the CH_3_ and NH_3_ groups change continuously (see Fig. 4(a)). However, the proton spectrum of the two peaks in (CH_3_NH_3_)_2_ZnCl_4_ at room temperature is recorded at chemical shifts of *δ* = 2.88 and 6.75. The ^1^H chemical shifts in (CH_3_NH_3_)_2_ZnCl_4_ are almost constant with temperature, as shown in Fig. 4(b).

The ^1^H spin–lattice relaxation times in the rotating coordinate frame of (CH_3_NH_3_)_2_MCl_4_ (M = Cu, Zn) were obtained for the CH_3_ and NH_3_ at several temperatures. The nuclear magnetization decay of ^1^H follows a single exponential function. Thus, *T*_1ρ_ can be determined by fitting the traces with the following equation:^35^

**Fig. 5:**
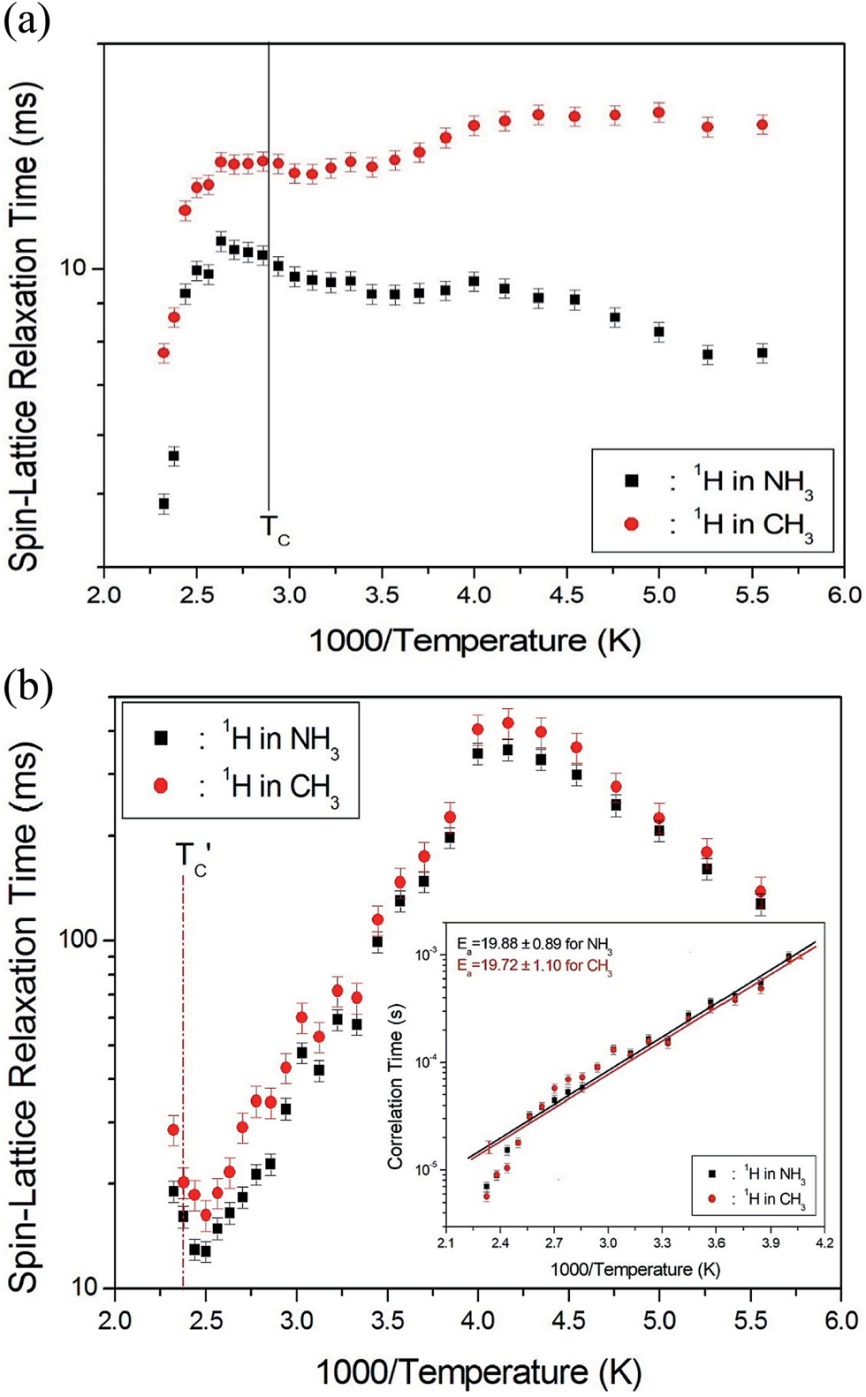
(a) ^1^H spin–lattice relaxation times in the rotating coordinate frame for CH_3_ and NH_3_ groups in (CH_3_NH_3_)_2_CuCl_4_ as a function of inverse temperature. (b) ^1^H spin–lattice relaxation times in the rotating coordinate frame for CH_3_ and NH_3_ groups in (CH_3_NH_3_)_2_ZnCl_4_ as a function of inverse temperature (inset: Arrhenius plots of the natural logarithm of the correlation time for each proton of CH_3_ and NH_3_ in (CH_3_NH_3_)_2_ZnCl_4_ as a function of inverse temperature).

1 *S*(*t*)/*S*(∞) = exp(−*t*/*T*_1ρ_),

where *S*(*t*) is the magnetization with the spin-locking pulse duration *t* and *S*(∞) is the total nuclear magnetization of ^1^H at thermal equilibrium. The values of ^1^H *T*_1ρ_ for two compounds in the rotating coordinate frame between 180 and 430 K are shown in Fig. 5(a) and (b) as a function of the inverse temperature. The *T*_1ρ_ values for the methyl protons and ammonium protons in the CH_3_NH_3_^+^ cations exhibit similar trends with temperature. The *T*_1ρ_ values of ^1^H in the CH_3_ and NH_3_ groups of (CH_3_NH_3_)_2_CuCl_4_ are almost continuous near *T*_C_, and these values are of the order of milliseconds. Above 400 K, the two *T*_1ρ_ values abruptly decrease, and the ^1^H *T*_1ρ_ values for CH_3_ are longer than those for NH_3_. In contrast, the significant change in the ^13^C *T*_1ρ_ values of (CH_3_NH_3_)_2_ZnCl_4_ is strongly affected, which is primarily considered the result of molecular motions. Further, the variation of *T*_1ρ_ with temperature exhibits a minimum of 16.3 and 12.8 ms for CH_3_ and NH_3_ near 400 K, respectively. This behavior of *T*_1ρ_ indicates that distinct molecular motions are present. It is clear that the minimum *T*_1ρ_ is attributable to the uniaxial rotation of CH_3_ and NH_3_ ions. The experimental value of *T*_1ρ_ is expressed in terms of the isotropic correlation time *τ*_C_ for molecular motion using the Bloembergen–Purcell–Pound (BPP) theory,^36^ according to which the *T*_1ρ_ value for a spin–lattice interaction of molecular motion is given by^37–39^

2 (*nT*_1ρ_^−1^) = 0.05(*μ*_o_/4π)^2^ (*γ*_H_*γ*_C_*ħ*/*r*_H–C_^3^)^2^[4*a* + *b* + 3*c* + 6*d* + 6*e*],

where *a* = *τ*_C_/[1 + *ω*_1_^2^*τ*_C_^2^], *b* = *τ*_C_/[1 + (*ω*_H_ − *ω*_C_)^2^*τ*_C_^2^], *c* = *τ*_C_/[1 + *ω*_C_^2^*τ*_C_^2^], *d* = *τ*_C_/[1 + (*ω*_H_ + *ω*_C_)^2^*τ*_C_^2^], and *e* = *τ*_C_/[1 + *ω*_H_^2^*τ*_C_^2^]. Here, *μ*_o_ is the permeability constant, *γ*_H_ and *γ*_C_ are the gyromagnetic ratios for the ^1^H and ^13^C nuclei, respectively, *n* is the number of directly bound protons, *r* is the H–C internuclear distance, *ħ* = *h*/2π (where *h* is Planck's constant), *ω*_H_ and *ω*_C_ are the Larmor frequencies of ^1^H and ^13^C, respectively, and *ω*_1_ is the spin-lock field of 55.55 kHz. Our data are analyzed assuming *T*_1ρ_ shows a minimum when *ω*_C_*τ*_C_ = 1 and the BPP relation between *T*_1ρ_ and *ω*_1_ is applicable. As the *T*_1ρ_ curves are found to exhibit minima, it was possible to determine the coefficient, 0.05(*μ*_o_/4π)^2^ (*γ*_H_*γ*_C_*ħ*/*r*_H–C_^3^)^2^, in the BPP formula. With this coefficient determined, we were then able to calculate the parameter *τ*_C_ as a function of temperature. The temperature dependence of *τ*_C_ follows a simple Arrhenius expression, *τ*_C_ = *τ*_Co_ exp(−*E*_a_/*RT*), where *τ*_Co_ is the pre-exponential factor, *T* is the temperature, *R* is the gas constant, and *E*_a_ is the activation energy. Thus, the slope of the straight-line portion of the semi-log plot can be used to determine *E*_a_. The activation energy for the uniaxial rotation of CH_3_ and NH_3_, obtained from the log *τ*_C_*vs.* 1000/*T* curve shown in the inset of Fig. 5(b), is 19.72 ± 1.10 and 19.88 ± 0.89 kJ mol^−1^, respectively, and is the same within the error range. In addition, the *E*_a_ value for CH_3_ and NH_3_ at temperatures below 200 K is 6.59 ± 0.51 and 5.92 ± 0.40 kJ mol^−1^, respectively.

**Fig. 6:**
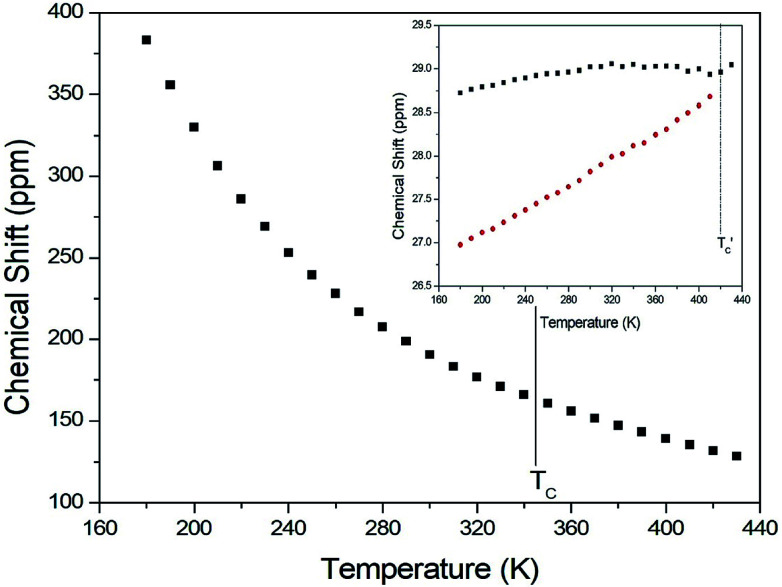
^13^C chemical shift in CH_3_ groups in (CH_3_NH_3_)_2_CuCl_4_ as a function of temperature (inset: that in (CH_3_NH_3_)_2_ZnCl_4_ as a function of temperature).

The chemical shifts for ^13^C in (CH_3_NH_3_)_2_CuCl_4_ were measured as a function of temperature, as shown in Fig. 6. At room temperature, the ^13^C CP MAS NMR spectrum shows a signal at a chemical shift of *δ* = 190.50 with respect to TMS. The ^13^C chemical shift slowly and monotonously decreases with temperature. In contrast, the chemical shifts for ^13^C in (CH_3_NH_3_)_2_ZnCl_4_ were also measured over the temperature range of 180 to 430 K, as shown in the inset of Fig. 6. At room temperature, the ^13^C CP MAS NMR spectrum possesses two signals at chemical shifts of *δ* = 27.82 and 29.02. These signals are attributed to the methyl carbons of the two inequivalent sites CH_3_ (1) and CH_3_ (2), and these results are consistent with the X-ray result previously reported:^18^ there exist two kinds of crystallographically inequivalent cations. The ^13^C chemical shifts near 426 K decrease by only one line; the change near 426 K was measured from the ^13^C chemical shift but not by the DSC result. Although the anomaly was not found around 426 K in the present DSC experiment, the existence of the ^13^C chemical shift and ^13^C *T*_1ρ_ was obtained. This anomaly near 426 K 
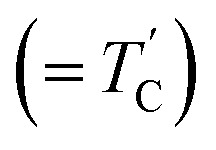
 is consistent with that obtained from Raman and IR spectra previously reported. There exist two kinds of inequivalent CH_3_ in (CH_3_NH_3_)_2_ZnCl_4_, whereas only one kind of equivalent CH_3_ in (CH_3_NH_3_)_2_CuCl_4_ exists. On the other hand, the chemical shifts of the CH_3_ groups in the ^13^C NMR spectra were very different between the two compounds. Generally, the paramagnetic contribution to the NMR shift is responsible for the NMR spectra.^40^ Thus, the ^13^C NMR chemical shift of (CH_3_NH_3_)_2_CuCl_4_, which contain paramagnetic ions, was significantly different from that of (CH_3_NH_3_)_2_ZnCl_4_, which does not contain paramagnetic ions. The differences in the ^13^C NMR chemical shifts could potentially be due to differences in the electron structures of the metal ions, in particular, the structure of the d electrons, which screen the nuclear charge from the motion of the outer electrons. Zn^2+^ has a filled d shell, whereas Cu^2+^ has one s electron outside the closed d shell.

**Fig. 7:**
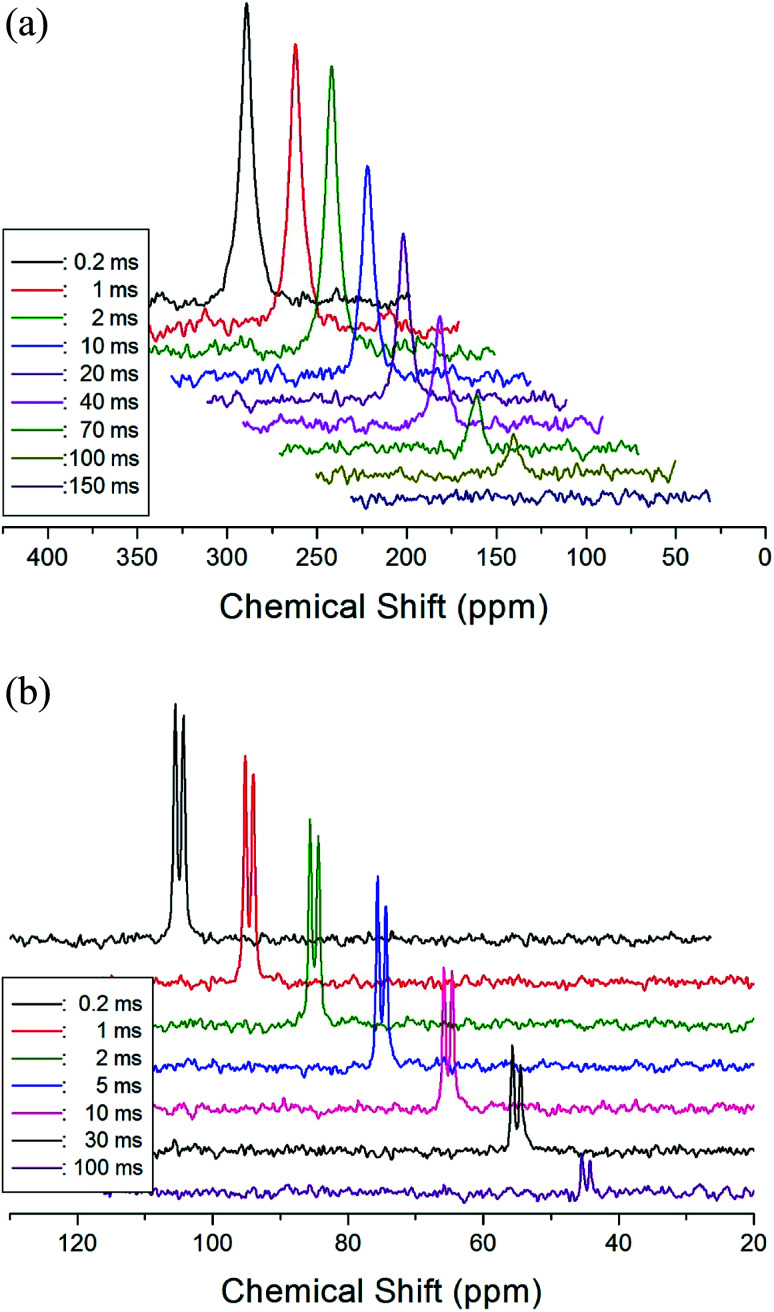
(a) Recovery spectra for delay times of ^13^C CP MAS NMR spectrum in (CH_3_NH_3_)_2_CuCl_4_ at room temperature. (b) Recovery spectra for delay times of ^13^C CP MAS NMR spectrum in (CH_3_NH_3_)_2_ZnCl_4_ at room temperature.

**Fig. 8:**
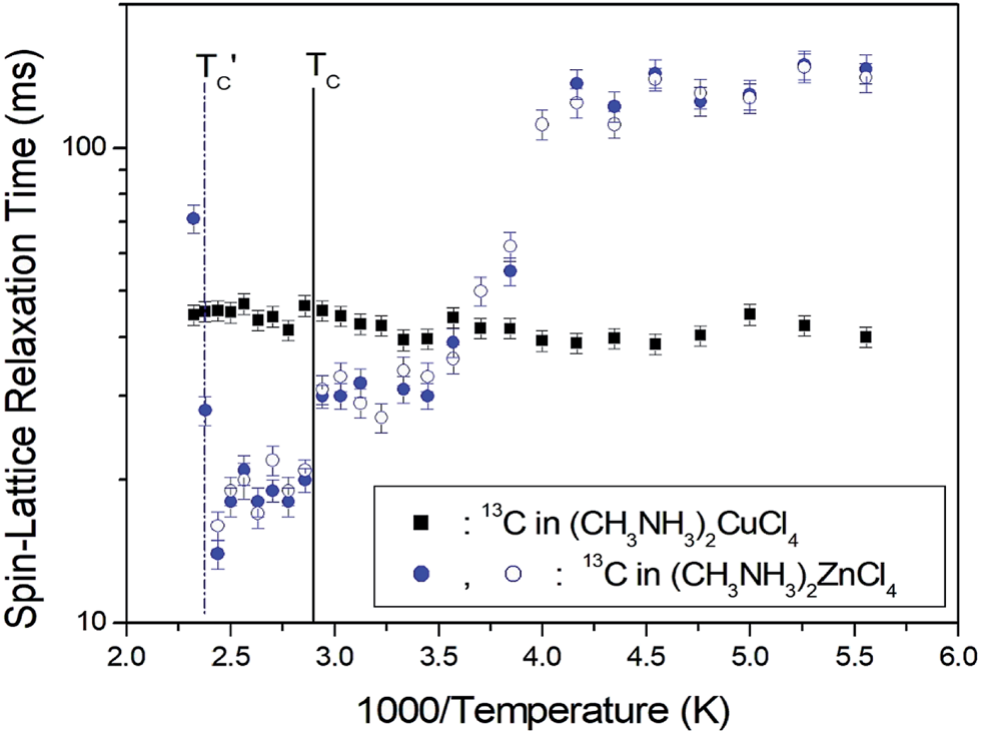
^13^C spin–lattice relaxation times in the rotating coordinate frame for CH_3_ groups in (CH_3_NH_3_)_2_CuCl_4_ and (CH_3_NH_3_)_2_ZnCl_4_ as a function of temperature.

The *T*_1ρ_ values were obtained for the carbon of (CH_3_NH_3_)_2_MCl_4_ (M = Cu, Zn) at several temperatures. ^13^C magnetization was generated by CP after spin-locking the protons. All magnetization traces obtained for the methyl carbon were described by a single exponential function *S*(*t*) = *S*(∞)exp(−*t*/*T*_1ρ_) of eqn (1).^35^ The recovery curves for various delay times of ^13^C in (CH_3_NH_3_)_2_CuCl_4_ and (CH_3_NH_3_)_2_ZnCl_4_ were measured at several temperatures. The saturation recovery traces for ^13^C were measured for delay times ranging from 0.2 to 150 ms at room temperature and are presented in Fig. 7(a) and (b). The recovery traces have different slopes at several temperatures. From these results, the *T*_1ρ_ values were obtained for the carbon in the two compounds as a function of the inverse temperature. The temperature dependence of the ^13^C *T*_1ρ_ values in (CH_3_NH_3_)_2_CuCl_4_ is illustrated in Fig. 8, and these values are almost constant with temperature. The *T*_1ρ_ values around *T*_C_ are unchanged, in agreement with the conclusion drawn from the ^13^C chemical shifts. In the case of (CH_3_NH_3_)_2_ZnCl_4_, the phase transition occurring at 
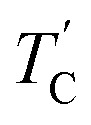
 (= 426 K) reported by Perez-Mato *et al.*^23^ is not observed from our DSC results, whereas the changes near 
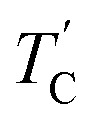
 are observed by the ^13^C chemical shift and ^13^C *T*_1ρ_ results. Thus, 
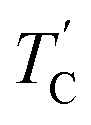
 is denoted by dotted lines in the inset of Fig. 5, 6, and 8. The *T*_1ρ_ values for the two ^13^C signals of CH_3_ (1) and CH_3_ (2) in (CH_3_NH_3_)_2_ZnCl_4_ are almost the same within the experimental error range.

**Fig. 9:**
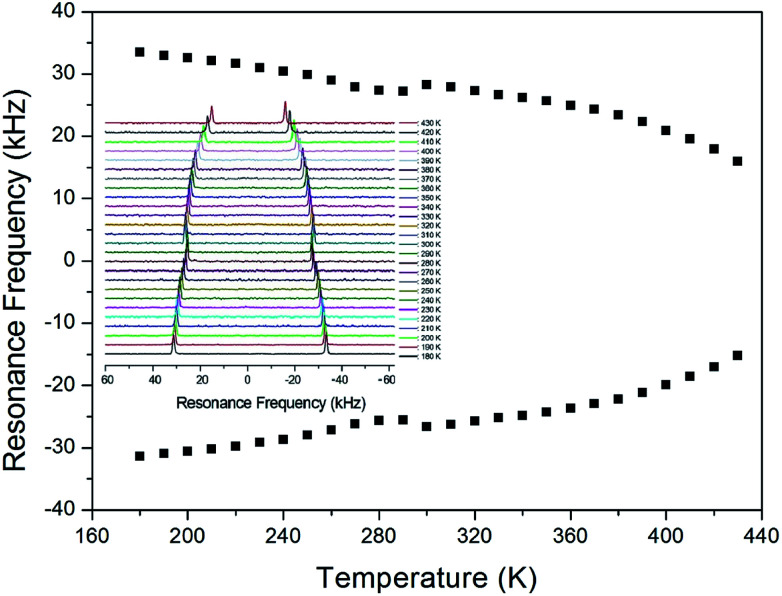
The temperature-dependent resonance frequency of ^14^N NMR spectra in (CH_3_NH_3_)_2_ZnCl_4_ single crystal as a function of temperature (inset: ^14^N NMR spectra as a function of temperature).

In order to obtain information concerning the possible distortion surrounding the ^14^N ion, the NMR spectrum of ^14^N (*I* = 1) in the laboratory frame was obtained using static NMR at a Larmor frequency of *ω*_0_/2π = 43.342 MHz. Two resonance signals were expected from the quadrupole interactions of the ^14^N nucleus. A magnetic field was applied along the crystallographic axis. The *in situ*^14^N NMR spectra and resonance frequency in (CH_3_NH_3_)_2_ZnCl_4_ single crystals are plotted in Fig. 9 as a function of temperature, respectively. The ^14^N NMR spectra of the two resonance signals for ^14^N are attributed to the NH_3_, and this splitting of the ^14^N resonance signals slightly decreases with temperature. The small change of the resonance frequency near 300 K is not related to the phase transition. Note that temperature-dependent changes in the ^14^N resonance frequency are generally attributed to changes in the structural geometry, indicating a change in the quadrupole parameter of the ^14^N nuclei. The electric field gradient tensors at the N sites vary, reflecting the changing atomic configurations around the nitrogen centers.

## 4. Conclusions

The ionic dynamics of (CH_3_NH_3_)_2_MCl_4_ (M = Cu, Zn), focusing on the role of the CH_3_NH_3_^+^ cation, were investigated by ^1^H MAS NMR, ^13^C CP MAS NMR, and ^14^N NMR as a function of temperature. We studied the molecular motions for ^1^H and ^13^C in (CH_3_NH_3_)_2_MCl_4_ (M = Cu, Zn) based on the spin–lattice relaxation time in the rotating coordinate frame. From the ^1^H *T*_1ρ_ results, we found that the molecular motions for ^1^H in (CH_3_NH_3_)_2_CuCl_4_ are very active at high temperatures. In addition, the activation energies for the uniaxial rotation of ^1^H for the CH_3_ and NH_3_ ions in (CH_3_NH_3_)_2_ZnCl_4_ have very similar values, and the uniaxial rotation occurs within the high-temperature range. The *T*_1ρ_ results reveal that the CH_3_ and NH_3_ cations exhibit high mobility at high temperatures. The *T*_1ρ_ provides insight into the changes in the cation reorientation rates induced by heating at high temperatures. On the other hand, the minima for (CH_3_NH_3_)_2_ZnCl_4_ are attributed to the uniaxial rotation of the CH_3_NH_3_ cations. However, minima such as *T*_1ρ_ for (CH_3_NH_3_)_2_CuCl_4_ were not reached for that compound. The lack of a minimum *T*_1ρ_ indicates that this motion is so slow that there was no detectable *T*_1ρ_ temperature dependence and also that the uniaxial rotation in (CH_3_NH_3_)_2_CuCl_4_ was slower than that in (CH_3_NH_3_)_2_ZnCl_4_. The motion of the CH_3_NH_3_ cations is slower than the C_3_ internal rotation of CH_3_ and NH_3_; therefore, it reveals *T*_1ρ_ minima in the high temperature regime above liquid nitrogen temperature. The minima related to the C_3_ rotation will appear in the low temperature regime.

A comparison with other compounds of the (CH_3_NH_3_)_2_MCl_4_ (M = Cu, Zn) indicates a different phase sequence for (CH_3_NH_3_)_2_MCl_4_ (M = Cd, Mn). For M = Cd, Mn, these systems at room temperature reveal orthorhombic symmetry followed by a tetragonal phase below room temperature. A phase with monoclinic symmetry is also reported at low temperatures. It is interesting to compare the results for (CH_3_NH_3_)_2_MCl_4_ with those for the analogous compounds containing other metals. In the case of (CH_3_NH_3_)_2_MnCl_4_ and (CH_3_NH_3_)_2_CdCl_4_, there is an intermediate tetragonal phase between the monoclinic and orthorhombic phases.^16,31^ In contrast, the phase transition sequence for (CH_3_NH_3_)_2_CuCl_4_ and (CH_3_NH_3_)_2_ZnCl_4_ changes to an orthorhombic to monoclinic structure with decreasing temperature.^22,31^

The created magnetization decay for each proton in (CH_3_NH_3_)_2_MCl_4_ (M = Cu, Zn) was analyzed by a single exponential function *S*(*t*)/*S*(∞) = *A* exp(−*t*/*T*_1ρ_), whereas that for each proton in (CH_3_NH_3_)_2_MCl_4_ (M = Mn, Cd) was analyzed by a double-exponential function *S*(*t*)/*S*(∞) = *A* exp(−*t*/*T*_1ρ_(s)) + *B* exp(−*t*/*T*_1ρ_(L)). These results are consistent with the interactions between the CH_3_NH_3_ cations and its surrounding MCl_4_^2−^ anions. This difference of *T*_1ρ_ is possibly due to the difference between the electron structures of metal ions. Cu^2+^ and Zn^2+^ have one and two s electrons, respectively, outside the closed d shell; Mn^2+^ has two s electrons in the unfilled 3d orbital; Cd^2+^ has two electrons outside the closed d shell.

The *T*_1ρ_ values for ^1^H of CH_3_ and NH_3_ indicate that the protons in the CH_3_NH_3_ cations that are involved in the hydrogen bonding exhibit large and small *T*_1ρ_ values corresponding to the long C–H and short N–H bonds, respectively. The molecular motion of the cation is induced by heating at high temperatures. The cation dynamics and interionic interactions through hydrogen bonds are expected to be closely related with the physical properties due to the potential applications. We will be examined the effect for lengths of alkyl chains as further study.

## Conflicts of interest

There are no conflicts to declare.

## Supplementary Material
